# The Influence of Kinesiophobia on Time to Clinical Recovery in Collegiate Athletes with Concussion

**DOI:** 10.1007/s40279-024-02144-8

**Published:** 2024-11-21

**Authors:** Daniel J. Rosenblum, Jacob E. Resch

**Affiliations:** Department of Kinesiology, University of Virginia, 550 Brandon Avenue, Charlottesville, VA 22903 USA

## Abstract

**Background:**

Several factors such as acute symptom severity, premorbid anxiety, and depression have been associated with concussion recovery. Elevated kinesiophobia has been associated with recovery from musculoskeletal conditions, as well as increased reaction time and vestibular–ocular motor dysfunction following concussion. However, kinesiophobia has yet to be evaluated as a modifier of concussion recovery time.

**Objectives:**

This study was designed to evaluate the role of acute kinesiophobia levels on days until clinical recovery in collegiate athletes with concussion. We hypothesized that collegiate athletes with elevated Tampa Scale of Kinesiophobia (TSK) scores would take a greater number of days to achieve clinical recovery compared with athletes with lower values.

**Methods:**

Division I collegiate athletes diagnosed with a concussion (*N* = 113, 19.9 ± 1.5 years, 42% female) participated in this descriptive laboratory study. Participants were assigned to high [≥ 37 (H-TSK, *n* = 54)] or low [< 37 (L-TSK, *n* = 59)] TSK groups on the basis of the first TSK values recorded within 72 h of their concussion. Participants were also administered the Revised Head Injury Scale (HIS-r) to assess symptom severity within 72 h of injury. The Immediate Postconcussion and Cognitive Test (ImPACT) battery was administered at baseline and used to gather demographic variables such as biological sex, age, history of anxiety/depression, and concussion history, and as part of the athletes’ symptom-free assessment. Days until clinical recovery between H-TSK and L-TSK groups were compared using a Mann–Whitney *U* test. Spearman’s rank correlation coefficients were calculated to determine the relationship between TSK and days until clinical recovery in addition to other modifiers of recovery. Multiple linear regression was used to evaluate days until clinical recovery as a function of the TSK total score, controlling for the HIS-r and ImPACT variables.

**Results:**

Days until clinical recovery was significantly longer in the H-TSK group (median difference = 2.5 days, *p* < 0.001) compared with the L-TSK group. A significant, moderate positive correlation between the TSK score and days to clinical recovery (*ρ* = 0.45, *p* < 0.001) was observed, which was also the strongest correlation among all variables. Our regression model demonstrated that for every point increase on the TSK, days until clinical recovery increased by 0.23 while controlling for total symptom severity, age, concussion history, psychiatric history, and biological sex (*β* = 0.23, *p* = 0.018). All other variables entered into the regression were not statistically significant.

**Conclusions:**

Our data suggest that athletes with TSK scores above 37 within 72 h of a concussion had a greater number of days until clinical recovery when compared with athletes with TSK values below 37. The TSK score had the highest correlation with days until clinical recovery when compared with other known modifiers of recovery, including total symptom severity. The TSK score was also the strongest predictor of days until clinical recovery. Collectively, these findings suggest that the TSK score should be considered by healthcare professionals to help inform effective management strategies for collegiate athletes with concussion.

## Key Points

**Table:** 

Collegiate athletes with clinically meaningful levels of kinesiophobia took longer to achieve clinical recovery as compared with those with lower levels.
The Tampa Scale of Kinesiophobia score was the strongest predictor of days until clinical recovery as compared with other modifiers of recovery such as acute symptom severity.
Clinicians should account for kinesiophobia in the acute phase of injury as this may help inform treatment strategies, such as physical activity and education, aimed at reducing fear of physical activity after concussion.

## Introduction

Sport-related concussion (SRC) may result in a variety of objective motor deficits, neurocognitive deficits, and increased symptom burden inclusive of anxiety and depression [1, 2]. The majority (80%) of collegiate athletes diagnosed with a SRC will recover within 2 weeks of their injury, which is considered a “typical” recovery [3, 4]. Approximately 15% of athletes with SRC experience symptoms beyond 30 days after injury, which is consistent with the newly defined persisting symptoms after concussion terminology [4–6]. While the most common predictor of prolonged recovery from SRC is having a greater number and severity of acute and subacute symptoms, [7] it has also been shown that athletes with preexisting or heightened levels of anxiety or depression may take longer to recover from a SRC compared with athletes without a prior history, and as such, psychological constructs should be accounted for when managing SRC [7].

A potential emerging psychological modifier of recovery from SRC may be kinesiophobia. Kinesiophobia has been previously defined as an excessive, irrational, and debilitating fear of physical movement and activity resulting from a feeling of vulnerability to painful injury or reinjury [8]. The construct of kinesiophobia was originally evaluated in patients with back pain [8, 9], but, the concept has been adopted in a variety of other conditions including, but not limited to, neck pain [10], fibromyalgia [11], and anterior cruciate ligament (ACL) injury [12, 13]. For example, Ardern and colleagues [13] concluded that the TSK score, among others, was a predictor of whether participants would return to their preinjury level of sport participation following ACL reconstruction. Additionally, Paterno et al. determined that patients with higher self-reported fear, measured via the TSK, were more likely to report lower levels of activity and to suffer a second ACL tear [12]. Other literature has demonstrated that kinesiophobia was one of the most common reasons for athletes to choose to not return to sport following ACL reconstruction [14].

Despite being widely examined in the musculoskeletal literature, kinesiophobia has not been as extensively investigated in the context of SRC. Reinking et al. [15] determined that adolescents (aged 12–18 years) with SRC reported higher TSK scores than healthy controls 2 weeks after their injury, with several injured participants exceeding the clinical threshold of 37, indicative of clinically meaningful kinesiophobia [16]. The authors also reported a moderate correlation between TSK scores and clinical reaction time for the concussion group at return to play (*r* = 0.50, *p* = 0.01) [15]. In a separate study, Anderson et al. concluded that high school athletes with high fear of re-injury were more symptomatic and more likely to exhibit vestibular/ocular motor symptoms over established clinical cutoffs [17]. Despite there being only a small number of studies in adolescent populations specifically, they do suggest that kinesiophobia may influence recovery from SRC, especially given its relation to symptom reporting in the aforementioned Anderson et al. study [17]. To date, despite prior evidence of kinesiophobia influencing recovery outcomes from other injuries, there is limited evidence examining kinesiophobia as a modifier of time to clinical recovery [18] following SRC in collegiate athletes. Therefore, the primary objective of this study was to evaluate whether kinesiophobia levels within 72 h of a diagnosed SRC influenced the time to clinical recovery in collegiate athletes, while controlling for other modifiers of recovery time such as age, concussion history, history of anxiety/depression, and acute symptom severity [7]. We hypothesized that following SRC, collegiate athletes with high levels of kinesiophobia would take longer to achieve clinical recovery than those with low levels.

## Methods

### Study Design

This was a prospective, descriptive cohort study. The primary independent variable was the composite TSK score of collegiate athletes within 72 h of a diagnosed concussion. The 72-h window was chosen due to prior work in animal models that demonstrated increased fear learning starting within 2 days after a concussive injury [19]. Additionally, work in human models has demonstrated elevated depression and mood disturbance within 3.56 ± 2.16 days of concussion in collegiate athletes [20]. Other work has shown mean anxiety/mood scores peak within 3 days after injury in collegiate athletes [21]. The primary dependent variable was days until clinical recovery. For this study, clinical recovery was defined as the difference, in days, between the date participants returned to baseline values on a multidimensional return to sport assessment and the date of injury.

### Patients or Other Participants

Participants in this study were NCAA division I collegiate athletes between 18 and 24 years of age who were part of an ongoing longitudinal investigation of SRC [22]. Data were collected between the 2020/21 and 2021/22 sport seasons. SRC was defined and diagnosed in accordance with the most recent International Conference on Concussion in Sport guidelines, which informed the university’s athletics department concussion protocol [2]. All participants suspected of having a concussion were immediately removed from play and were diagnosed by a certified and licensed athletic trainer or physician [2]. This study was approved by the University of Virginia’s institutional review board (IRB#: 20052) and all participants provided informed consent prior to data collection.

### Measures

The ImPACT (ImPACT Applications, Pittsburgh, PA) is the most commonly used computerized neurocognitive test (CNT) used by surveyed certified athletic trainers [23]. The ImPACT battery consists of self-reported medical history, a symptom scale, and a variety of neurocognitive tests. The ImPACT was administered upon each athlete’s entrance into the university’s athletic department in alignment with a NCAA-approved concussion management protocol. In addition to the ImPACT, as part of the multidimensional baseline and post-injury assessments, the Sensory Organization Test, Vestibular/Ocular Motor Screen, Timed Tandem Gait, Revised Head Injury Scale, Generalized Anxiety Index-7, and Patient Health Questionnaire-9 were also administered. The Tampa Scale of Kinesiophobia was only administered after injury. For this study, the medical history domain of the baseline ImPACT medical history was reviewed to record history of prior diagnosed concussion, history of anxiety/depression, history of ADD (attention-deficit disorder)/ADHD (attention-deficit hyperactivity disorder), and biological sex. Specific to concussion history, participants were asked only to report those concussions that were medically diagnosed by a healthcare professional.

The Revised Head Injury Scale (HIS-r) is a symptom inventory that consists of 22 symptoms related to SRC [24]. The participant is asked to rate the duration and severity of each symptom during the preceding 24 h on a Likert scale. Duration consists of 1 (brief) to 6 (consistent) and severity consists of 0 (not severe) to 6 (severe). Sensitivity and specificity have been previously reported in collegiate athletes to be 77.5% and 100%, respectively [25]. Symptom severity was determined through the summation of the severity section of the HIS-r. The HIS-r was administered daily after concussion by each participants’ athletic trainer. Upon reporting 0 symptoms on the HIS-r, all participants were administered a multidimensional return to play assessment.

The TSK consists of 17 items in which participants self-report their kinesiophobia levels on a four-point Likert scale from “strongly disagree” to “strongly agree” and is the most commonly used tool for measuring kinesiophobia [26]. The TSK has been previously demonstrated to have strong test–retest reliability up to 29 days following the first administration (*r* = 0.78–0.90) and to have good internal consistency (Cronbach’s *α* = 0.76–0.79) [27, 28]. The TSK has also been demonstrated to have acceptable face, construct, and content validity in patients with chronic low back pain [29]. The TSK composite score can range from 17 to 68, with higher scores indicating higher (adverse) levels of kinesiophobia. Items 4, 8, 12, and 16 are inversely scored to minimize the influence of erroneous reporting of kinesiophobia [8, 27]. For the purposes of this study, any participant with a score of ≥ 37 was considered to have “high” kinesiophobia (H-TSK), while anyone with scores < 37 was considered to have “low” kinesiophobia (L-TSK) [16, 17].

### Procedures

Upon entrance to the university, all participants were administered the multidimensional baseline assessment inclusive of computerized measures of balance and neurocognitive function as well as patient reported outcomes by a trained study team member. Upon the diagnosis of a concussion, participants were administered the HIS-r followed by the TSK within 72 h of their injury by their certified athletic trainer. Participants were excluded from our analyses if the first administration of either questionnaire occurred beyond 72 h of injury. Examples for exclusion included if an athlete did not disclose symptoms within the 3-day period or in the event of missing data. Following the initial assessment, participants were administered the HIS-r daily by their individual athletic trainer until 0 symptoms were endorsed. Participants were then readministered the multidimensional assessment by a trained study team member to determine their readiness for return to sport. Once the participants returned to their baseline values on all clinical measures, they were considered to have achieved clinical recovery, and then were ultimately progressed through a graded return to sport physical activity protocol consistent with international guidelines [2].

### Statistical Analyses

To compare H-TSK versus L-TSK groups, continuous demographic data were evaluated using independent samples *t* tests while categorical demographic data (i.e., sex, anxiety/depression history, and concussion history), by TSK group, were evaluated using chi-squared (*χ*^2^) analysis. Due to 4/5 of our primary outcome variables (age, days to symptom resolution at rest, concussion history, and HIS-r total severity) violating the Shapiro–Wilk test of normality (all four, *p* < 0.001), we elected to use non-parametric statistics for data analysis. Non-normally distributed data are presented as median [interquartile range (IQR)]. Spearman’s rank correlation coefficient (rho) was used to investigate any correlation between the TSK and days to clinical recovery. Correlation coefficients were interpreted as 0.00–0.10 (negligible), 0.10–0.39 (weak), 0.40–0.69 (moderate), 0.70–0.89 (strong), and 0.90–1.00 (very strong) [30]. Mann–Whitney *U* testing was used to compare days to clinical recovery between each TSK group. Multiple linear regression evaluated differences in days to clinical recovery as a function of the TSK total score while holding age, anxiety/depression history, concussion history, HIS-r total symptom severity (within 72 h of diagnosis), and sex constant. All analyses were performed in the statistical software *Jamovi* version 2.3.21.0 with *α* = 0.05.

## Results

**Fig. 1:**
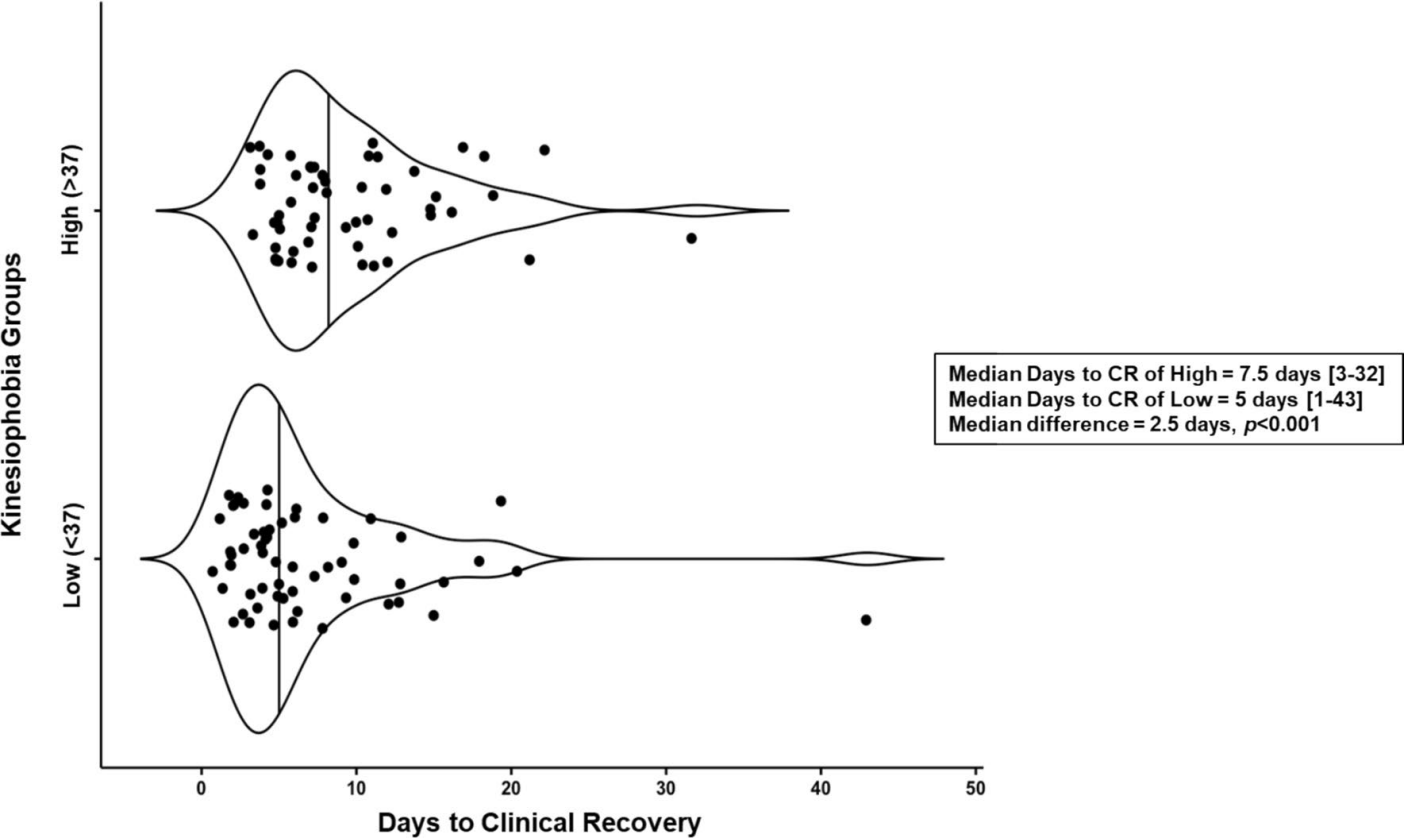
Violin plots demonstrating relationship between Tampa Scale of Kinesiophobia group medians and days to clinical recovery (CR)

**Fig. 2:**
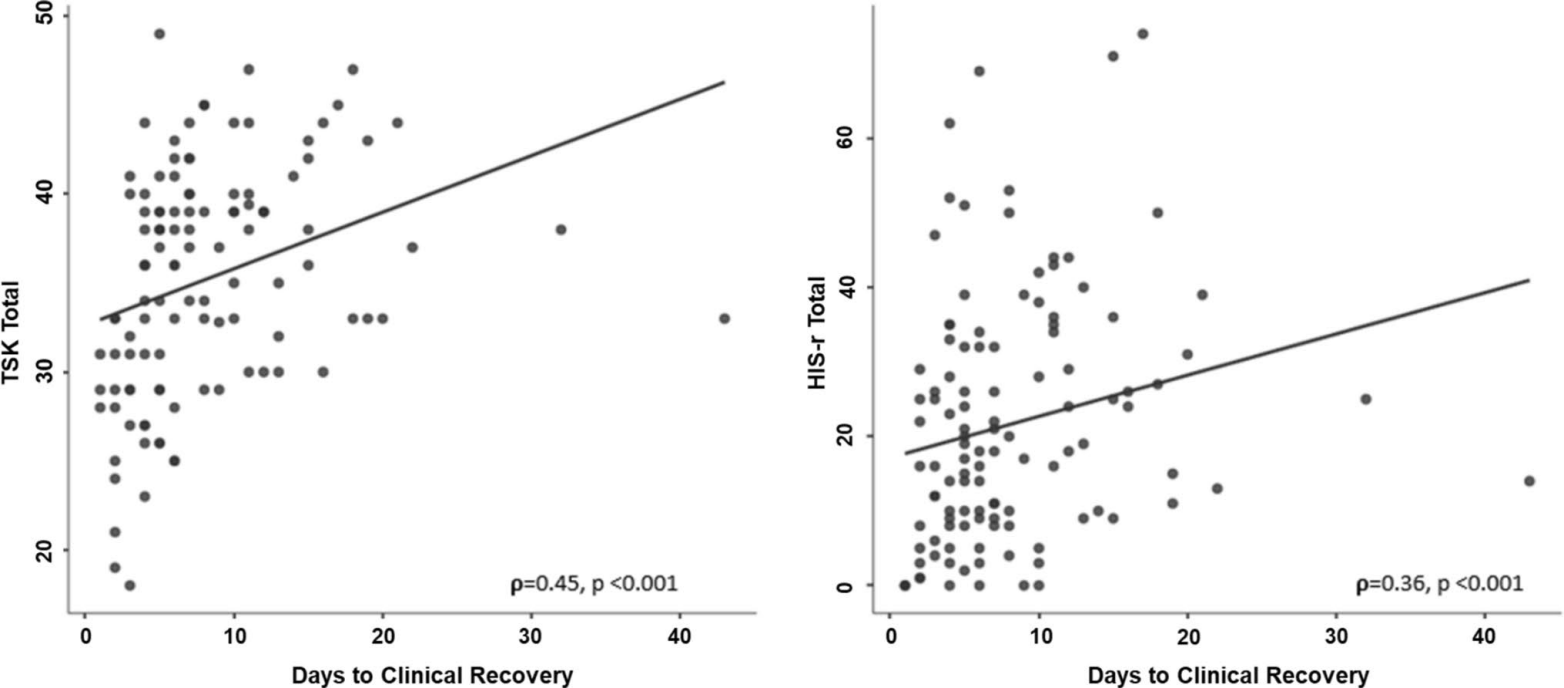
Correlations between Tampa Scale of Kinesiophobia (TSK) Total, Revised Head Injury Scale (HIS-r) severity, and days to clinical recovery

A total of *N* = 113 (19.9 ± 1.5 years, 42.3% female) collegiate athletes with a diagnosed SRC participated in our study. Descriptive statistics between each group (H-TSK, L-TSK) can be found in Table 1. Groups were similar in terms of age, concussion history, and previous diagnosis of ADD/ADHD (*p* < 0.05). A greater proportion of female participants reported H-TSK [28/48 (58.3%)] than male participants [26/65 (40%), *χ*^2^_(1)_ = 3.72, *p* = 0.054] although this difference was not statistically significant. There was a greater proportion of individuals with a previous diagnosis of anxiety/depression in the H-TSK group [16/54 (29.6%) H-TSK versus 7/59 (11.8%) L-TSK, *χ*^2^_(1)_ = 5.49, *p* = 0.019]. Of those with anxiety/depression histories, 16/23 (69.5%) were in the H-TSK group. Sport type between groups can be found in Table 2. Sport type was defined similar to that of Meehan et al. [31]. Overall, the median time to clinical recovery was 6 (4–11) days. There was a statistically significant difference in days to clinical recovery between groups; the H-TSK group took longer (median difference = 2.5 days, *U* = 946.5, *p* < 0.001; Fig. 1). A significant and moderate positive correlation was observed between the total TSK score and days until clinical recovery (*ρ* = 0.45, *p* < 0.001) while a significant and weak correlation was observed for the HIS-r total and total TSK score (*ρ* = 0.33*, p* < 0.001). There was also a significant and weak correlation between the HIS-r total and days until clinical recovery (*ρ* = 0.36, *p* < 0.001) (Table 3 and Fig. 2).

**Table 1:** Descriptive statistics. Categorical variables are presented as number (percentage of group). Continuous variables are presented as median [IQR] due to non-normally distributed data

Variable	H-TSK (*n* = 54)	L-TSK (*n* = 59)	Overall (*N* = 113)
Age (y)	19.0 [1.0]	20.0 [2.0]	19.0 [2.0]
Sex (female)	28 (58%)	20 (42%)	48 (42%)
ADD/ADHD	9 (16%)	8 (13%)	17 (15%)
Anxiety/depression (yes)^a^	16 (29%)	7 (12%)	23 (20%)
Concussion history (yes)	23 (43%)	23 (39%)	46 (41%)
Concussion history (continuous)	0 [1], range 0–4	0 [1], range 0–3	0 [1], range 0–4
White	38 (64%)	42 (78%)	80 (71%)
Black or African/American	8 (15%)	10 (17%)	18 (16%)
Hispanic or Latino	3 (6%)	2 (3%)	5 (4%)
Native Hawaiian or other Pacific Islander	1 (2%)	2 (3%)	3 (3%)
Asian	0 (0%)	2 (3%)	2 (2%)
Multi race/ethnicity	3 (6%)	0 (0%)	3 (3%)
Other race/ethnicity	1 (2%)	1 (2%)	2 (2%)

*H-TSK* High Tampa Scale of Kinesiophobia, *L-TSK* Low Tampa Scale of Kinesiophobia, *HIS-r* Revised Head Injury Scale, *ADD/ADHD ADD* attention-deficit disorder)/attention-deficit hyperactivity disorder

^a^Denotes statistical difference between groups (chi-squared for categorical, Mann–Whitney *U* for continuous)

**Table 2:** Sport type per group, presented as number (percentage of group)

Group	Non-contact	Contact	Collision
H-TSK (*n* = 54)	14 (26%)	22 (41%)	18 (33%)
L-TSK (*n* = 59)	9 (15%)	16 (27%)	34 (58%)
Overall (*N* = 113)	23 (20%)	38 (34%)	52 (46%)

*H-TSK* High Tampa Scale of Kinesiophobia, *L-TSK* Low Tampa Scale of Kinesiophobia

**Table 3:** Spearman’s correlation coefficient matrix

Variable		Days to CR	TSK total	HIS-r severity	Concussion history
Days to CR	Spearman's rho *p* value	– –			
TSK total	Spearman's rho *p* value	0.45* < 0.001	– –		
HIS-r total	Spearman's rho *p* value	0.36* < 0.001	0.33* < 0.001	– –	
Concussion history	Spearman's rho *p* value	− 0.09 0.35	− 0.04 0.68	0.02 0.82	– –
Age	Spearman’s rho *p* value	− 0.13 0.15	− 0.16 0.26	− 0.05 0.57	0.19* 0.03

*TSK* Tampa Scale of Kinesiophobia, *HIS-r* Revised Head Injury Scale, *CR* clinical recovery

*p < 0.05

With regards to the multiple linear regression model, for every point increase on the TSK, time to clinical recovery increased by 0.23 days while controlling for age, anxiety/depression history, concussion history, HIS-r total symptom severity, and sex (*β*_TSK_Total_ = 0.23, *p* = 0.018; Table 4).

**Table 4:** Multiple linear regression model

Predictor	Coefficient	Std. error	*t* value	*p* value	Adj *R*^2^
Intercept	− 0.31	9.08	− 0.04	0.972	0.09
TSK total	0.23	0.09	2.39	0.018*	
HIS-r total	0.04	0.04	1.18	0.241	
Concussion history	− 0.43	0.71	− 0.60	0.547	
Anxiety/depression_(yes)_	2.41	1.47	1.65	0.103	
Sex_(female)_	0.58	1.19	0.49	0.628	
Age	− 0.05	0.40	− 0.13	0.899	

*TSK* Tampa Scale of Kinesiophobia, *HIS-r* Revised Head Injury Scale

*Denotes statistical significance at *p* < 0.05

## Discussion

Kinesiophobia is an emerging concept as it relates to SRC recovery. While there are several other modifiers of recovery from a SRC, our study suggests that kinesiophobia may also play a prognostic role in terms of days until clinical recovery for collegiate athletes. The primary aim of this study was to determine the influence of acute kinesiophobia on time until clinical recovery in collegiate athletes diagnosed with SRC. Our results supported our hypothesis that athletes who reported levels of kinesiophobia above a previously established clinical cutoff of ≥ 37, as measured by the TSK within 72 h of SRC, took longer to achieve clinical recovery. In fact, the H-TSK group achieved symptom resolution approximately 2.5 days later compared with those in the L-TSK group. Interestingly, TSK scores were also both the strongest predictor of and more strongly correlated with days to clinical recovery than other commonly accepted modifiers of recovery such as acute symptom burden. We also observed that a premorbid diagnosis of anxiety/depression may influence TSK reporting behavior.

Our findings are in alignment with related research that suggests kinesiophobia may associate with SRC [15, 17, 32]. Specifically, Reinking et al. reported that kinesiophobia was moderately correlated with clinical reaction time (*r* = 0.50, *p* = 0.01) in that as kinesiophobia increased, reaction time slowed in adolescent athletes. Additionally, at their RTP assessment, 28% of the injured adolescent athletes in that study continued to report high levels of kinesiophobia [15]. This, however, was not a significant difference compared with the 24% of controls who also reported high levels at the same timepoint. Anderson et al. [17] evaluated the influence of fear of re-injury, as measured by the TSK, on certain recovery outcomes such as the ImPACT battery, a symptom inventory, the Vestibular/Ocular Motor Screen, and days to recovery in high school athletes. While they defined a higher score on the TSK as an indication of greater fear of re-injury, it is important to clarify that the TSK total score is not solely related to fear of re-injury. Prior research has utilized confirmatory factor analyses of the TSK and suggests that the most common factors associated with the TSK include activity avoidance, pathological somatic focus, and harm, with only one study suggesting fear of re-injury as one of four factors [29]. Nevertheless, Anderson et al. concluded that high school athletes in the high fear group reported statistically significant increases in total, cognitive–migraine–fatigue, and affective symptoms [17]. Participants in the high fear group were also observed to be five times more likely to have Vestibular Ocular/Motor Screen values indicative of vestibular/ocular dysfunction when compared with those in the low fear group [17]. However, both groups had similar recovery times.

In contrast to Anderson et al. [17], our results suggest that collegiate athletes with high levels of acute kinesiophobia took longer to achieve clinical recovery than those with low levels. A potential rationale for this discrepancy is timing of the TSK administration and sample size. Anderson and colleagues administered the TSK on average 5 days after the injury, while we administered it within 3 days [17]. In addition, Anderson et al. reported total recovery time (i.e., total number of days from the date of injury to medical clearance, inclusive of asymptomatic physical and cognitive activity), rather than just the clinical recovery timepoint used in this study, exclusive of asymptomatic physical activity [17].

A strength of the current study is its relatively large sample size as compared with related studies [15, 17, 32]. While we cannot conclude from our study the definitive role of kinesiophobia in recovery from SRC, it is plausible that it is a modifier of time to clinical recovery in collegiate athletes. This is especially reasonable given the moderate correlation and statistically and clinically significant median differences that we observed. From a clinical standpoint, a difference in 2.5 days may be the difference between an athlete participating in a competition or not after completing a return to sport protocol. Similarly, additional interventions following concussion including prompt removal from sport [44] and subsymptom threshold exercise [36] have reported comparable improvements in recovery time. While more work surrounding the prevention of delayed recovery after concussion is warranted, clinicians should account for psychological variables such as kinesiophobia, specifically during the acute phase of injury. Doing so may provide clinicians with multiple points of intervention to ultimately afford their patient the opportunity to have a more typical recovery.

While an intervention for kinesiophobia in the context of concussion has yet to be fully identified, it may be as simple as providing the injured athlete opportunities to gain confidence in their physical abilities through physical activity and conversations with sport psychologists. Exposure therapy is one of the most effective ways to treat phobia-related disorders [33]. For athletes with concussion, this may be done with exposure to prescribed physical activity. Prescribed physical activity has been shown to reduce kinesiophobia in variable populations [26]. Smulligan et al. demonstrated that greater reductions in TSK scores were moderately correlated with higher daily step counts and exercise frequency among children with persisting symptoms (≥ 28 days) [34]. Given these findings, we hypothesize that reductions in kinesiophobia may also be a partial explanation of why subsymptom threshold aerobic exercise following concussion reduces recovery time. As such, it is important for clinicians to consider the implications of prescribing complete physical rest after an “invisible” injury, such as concussion, and the implications it may have on athlete psychology. By prescribing complete rest, athletes may internalize the prescription by viewing physical activity as bad, resulting in a fear of physical activity, especially given our finding that a premorbid diagnosis of anxiety/depression can influence TSK reporting behavior. While adhering to physical rest, athletes may then catastrophize their lingering symptoms, which may lead to misattribution and kinesiophobia as evidenced by the fear avoidance model [35]. As the breadth of concussion literature supports an active as opposed to passive approach to rehabilitation [36, 37], the evaluation of kinesiophobia acutely after injury may be more relevant than ever.

Our findings suggest that kinesiophobia, as measured by the TSK, may be a modifier of recovery from concussion, and even more so than commonly accepted modifiers of recovery such as acute symptom burden and anxiety/depression. Although a prior meta-analysis (unrelated to concussion) found strong associations between pain-related fear and disability (i.e., occupational, social, or recreational), this relationship was not moderated by pain characteristics such as duration and intensity [38]. In the context of concussion, these pain characteristics can be contextualized to include deficits such as symptom severity and duration. We observed that kinesiophobia can influence recovery time separately from symptom severity after concussion. Another intriguing finding of prior work was the existence of a strong relationship between pain-related fear and disability among people experiencing acute pain [38]. The authors concluded that future work should evaluate how reducing acute pain-related fear may impede progression to chronic pain [38]. In the context of those findings, in combination with the findings of our study, kinesiophobia may be an important and measurable signal for developing prolonged symptomology. If clinicians do not evaluate kinesiophobia during the acute phase of injury, they may omit an opportunity to intervene and facilitate a more typical recovery from concussion. It is important to note that a “quicker” recovery is not always a “better” recovery; however, we feel that accounting for a more holistic view of an athlete after concussion may result in the identification of multiple opportunities for intervention as opposed to solely characterization. The identification of one or more therapeutic targets may assist athletes to achieve a more typical and safe return to sport. The latter is particularly important as evidence supports a relationship between return to sport after concussion and an increased risk of subsequent musculoskeletal injury [39, 40].

A systematic review [39] of 13 articles explored this relationship and concluded that 4 studies showed an increased risk of subsequent lower extremity injury within 90 days following a diagnosed concussion. In the same review, six studies demonstrated an elevated risk of subsequent injury within 1 year of concussion [39]. The underlying mechanism(s) for the relationship between concussion and lower extremity injury has/have yet to be determined. Previous literature examining ACL injury has suggested that kinesiophobia can negatively influence biomechanics and therefore risk of injury [12, 41]. Specific to concussion, Thompson et al. concluded that there was a greater proportion of athletes who reported kinesiophobia levels above 37 at their return to play assessment who were subsequently diagnosed with a time-loss musculoskeletal injury within 180 days of returning to play compared with those who scored below 37 [32]. Moreover, it is plausible that decreased reaction time, as a function of kinesiophobia [15], could increase an athlete’s risk of sustaining a subsequent injury [32], especially given findings that reaction time deficits can persist up to 59 days post-injury [42]. This evidence remains mixed, however, with one study concluding that clinical reaction time was not a predictor of subsequent musculoskeletal injury in collegiate athletes [43]. This interpretation provides an initial framework for establishing kinesiophobia not only as a modifier of recovery, but also as a potential mechanism for influencing risk of subsequent musculoskeletal injury following return to sport after a SRC.

Our study had limitations. First, the TSK clinical cutoff scores used in this study were established in a population of patients with low back pain; thus it is important to note that the TSK has not been validated specifically in collegiate athletes with concussion. However, we also treated the TSK as a continuous variable in our analyses and continued to observe an association between the TSK score and symptom resolution time. Additionally, despite not being designed for concussion, the TSK is agnostic to a specific injury. For example, many questions ask about “My pain… or “My medical condition… or “My accident…” etc. We did not alter the verbiage of the TSK to reflect a different injury, which we believe supports the original measurement properties. It is imperative that future studies assess the testing properties of the TSK to support or to establish new clinical cutoffs specifically in athletes diagnosed with SRC. Additionally, our participants self-reported their medical history, which may be subject to recall bias. It is also plausible that some athletes may have received specific interventions such as medication or physical therapy that may have contributed to symptom status. This was not recorded in this study. Lastly, all participants’ symptoms resolved within a relatively typical time frame [4]. Despite this, those in the H-TSK group still took longer to achieve clinical recovery.

## Conclusions

The results of our study demonstrate that collegiate athletes with high levels of kinesiophobia assessed within 72 h of concussion may take longer to achieve clinical recovery compared with athletes with low levels of kinesiophobia. Additionally, a premorbid diagnosis of anxiety/depression may influence TSK reporting behavior. Clinicians should consider measuring kinesiophobia following concussion to identify collegiate athletes who may benefit from early psychological or physical interventions designed to reduce fear of movement prior to returning to play to foster a more typical recovery following SRC.

## Declarations

### Data availability

All data are available upon request from qualified individuals.
